# Supplementary material for: How lasing happens in CsPbBr3 perovskite nanowires
Source: Nat Commun. 2019 Jan 16;10:265. doi: 10.1038/s41467-018-07972-7 (PMC6335413; doi:10.1038/s41467-018-07972-7)
Supplement: Supplementary file 1 — Supplementary Information [file 41467_2018_7972_MOESM1_ESM.pdf]

Supplementary Information

How Lasing Happens in $CsPbBr_3$ Perovskite Nanowires

Schlaus et al.

How Lasing Happens in $CsPbBr_3$ Perovskite Nanowires Supplementary Information

Andrew P. Schlaus, Michael S. Spencer, Kiyoshi Miyata, Fang Liu, Xiaoxia Wang, Ipshita Datta, Michal Lipson, Anlian Pan, X-Y. Zhu

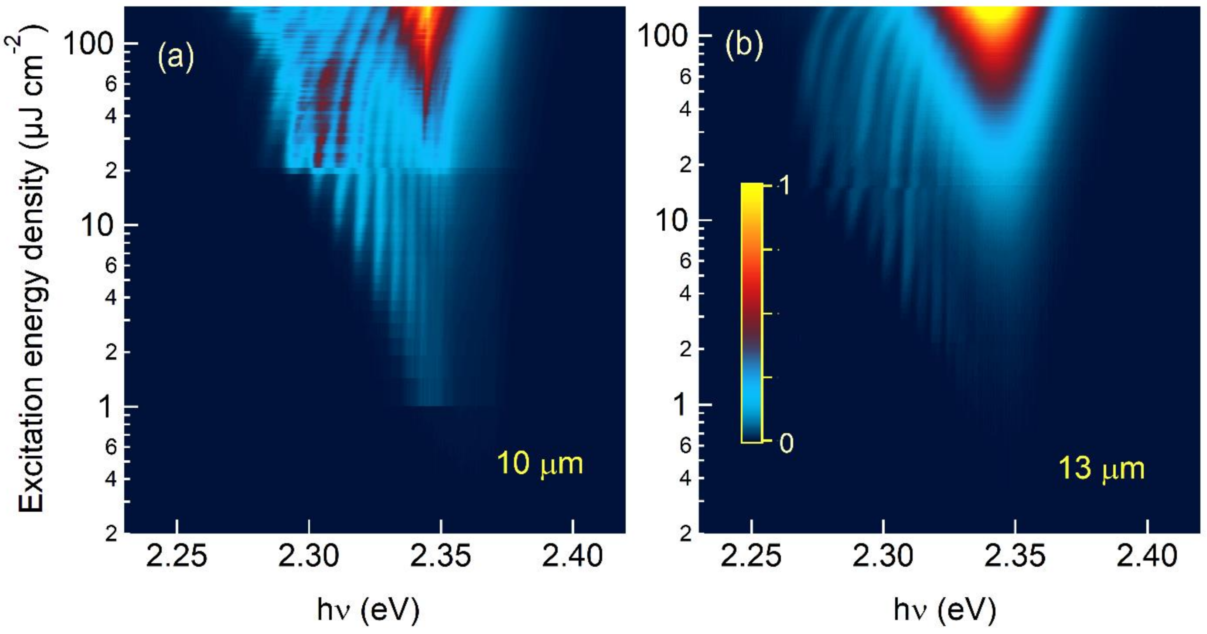

Supplementary Figure 1. Excitation density dependent lasing spectra of two nanowires in the lasing and saturation regions. The lengths of the nanowires are (a) 10 µm and (b) 13 µm. All spectra obtained under the same conditions as those in the main text. Each sample was uniformly excited at hν = 3.0 eV at a substrate temperature of 80 K.

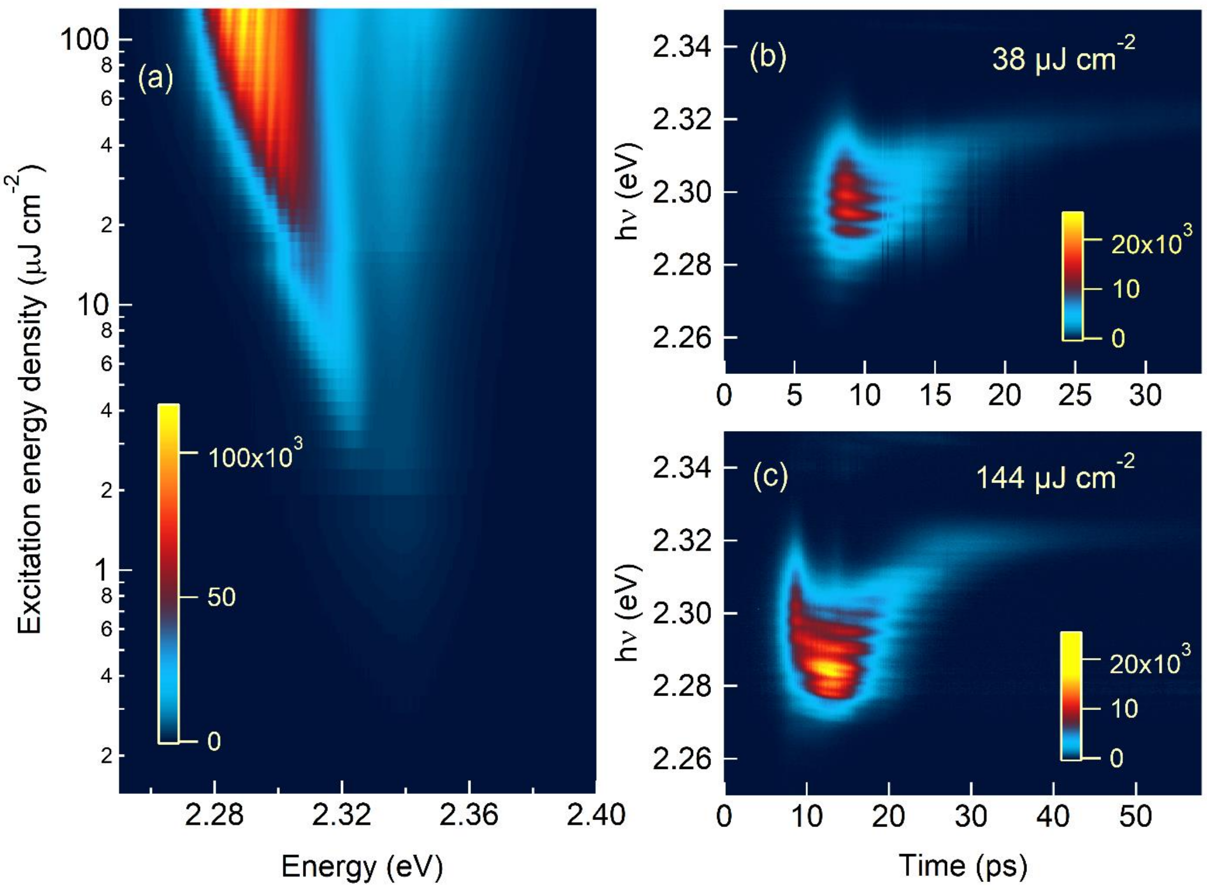

Supplementary Figure 2. Excitation density dependent and time-resolved lasing spectra of a microplate (3.5 x 13μm). (a) Time-integrated emission spectrum as a function of excitation energy density. (b-c) Time-resolved emission spectrum at excitation energy densities of ρ = 38 and 144 μJ cm^{-2}. All spectra obtained under the same conditions as those in the main text. Each sample was uniformly excited at hν = 3.0 eV at a substrate temperature of 80 K.

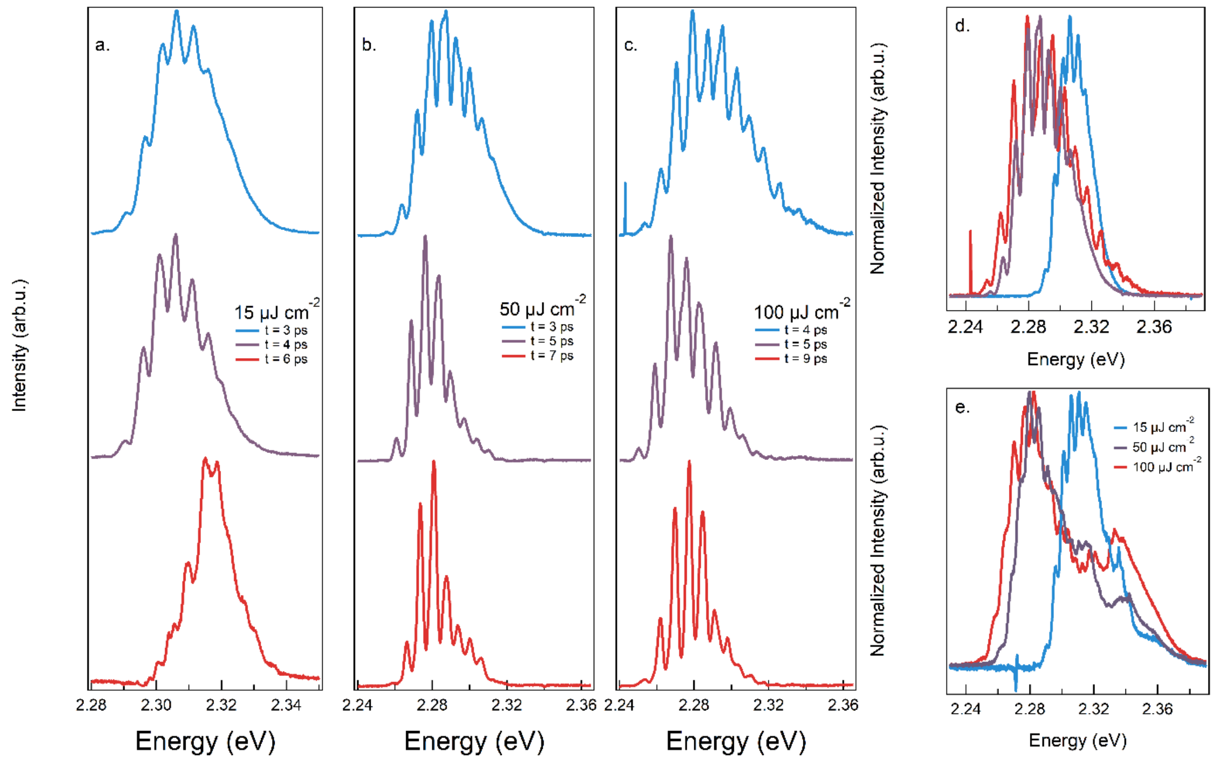

Supplementary Figure 3. Sample lasing spectra at selected delay times and excitation densities. Panels a,b,c display line cuts at three excitation densities from the 2D pseudo color plots in Fig 3 in the main text. The first line cut is taken at delay time when the spectrum is broadest, with subsequent line cuts demonstrating spectral narrowing. The top line cuts from a,b, and c are displayed on top of each other in d for comparison. Panel e shows time-integrated spectra from the three sets of time-resolved spectra.

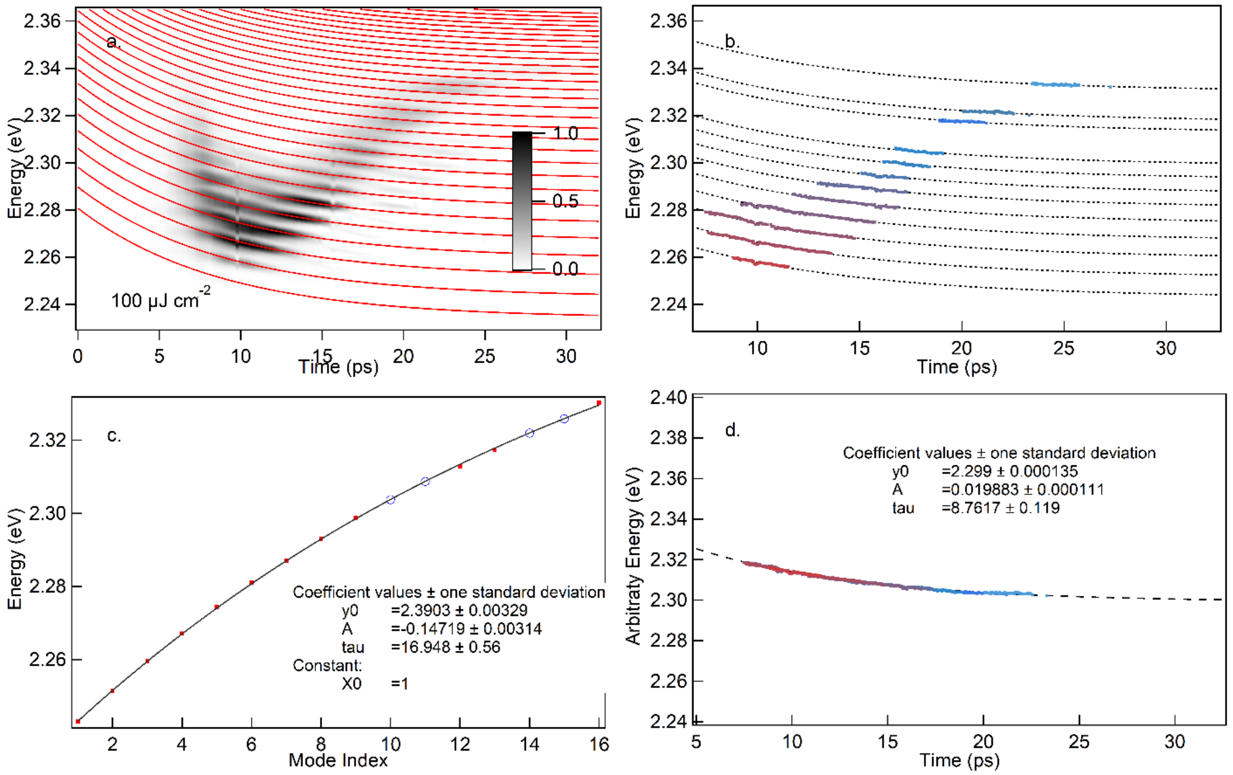

Supplementary Figure 4. Extracting laser gain profiles and mode energies. (a) Overlay of the numeric temporal photonic mode profiles with the lasing spectrum at 100 μJ cm^{-2} excitation energy density. (b) Results of the numeric image analysis where the coloured curves indicate the resulting traces of the numeric image analysis, along with the black dashed curves which represent the fit of their temporal profile based off the global fit done in (d). (c) Fit of the free spectral range in the energy region around the gain profile. (d) Numerically extracted lasing mode energies from all the lasing modes (color dots), with the time evolution of each mode shifted horizontally until all data sets fall on the same curve. Globally fit to all the data points is shown as the dashed curve.

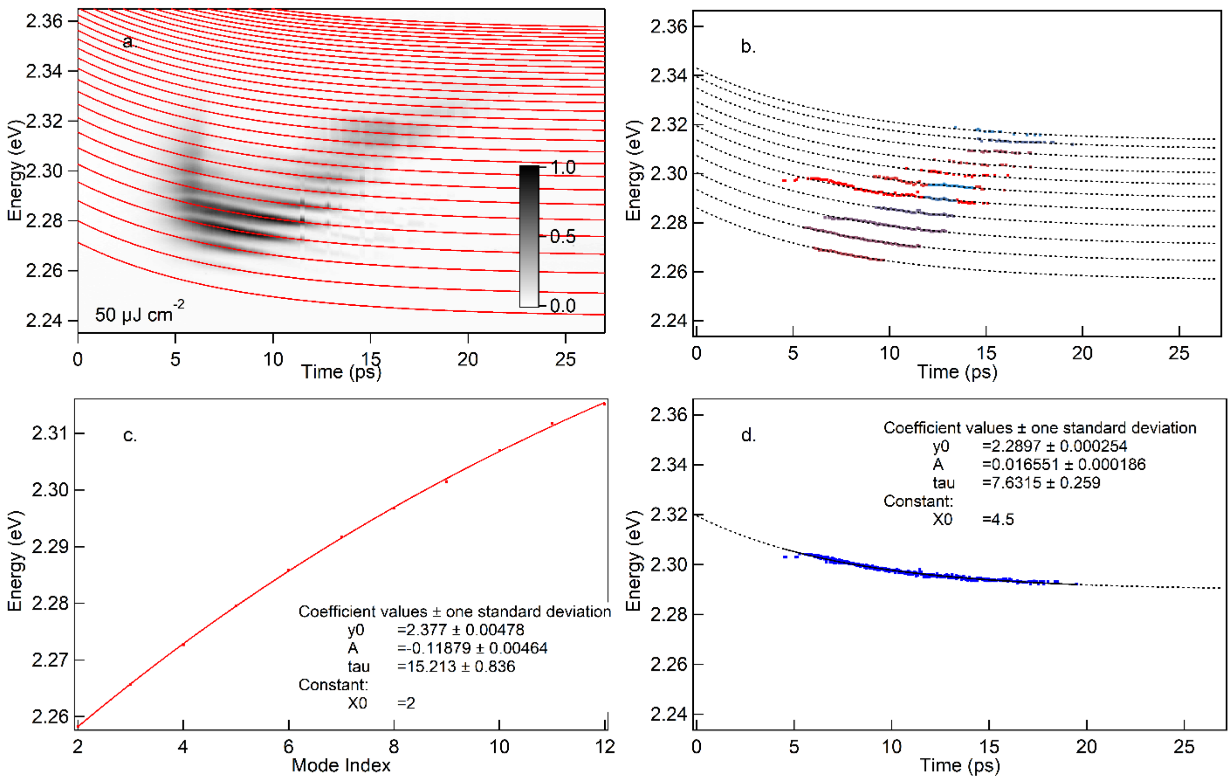

Supplementary Figure 5. Extracting laser gain profiles and mode energies. (a) Overlay of the numeric temporal photonic mode profiles with the lasing spectrum at 50 μJ cm^{-2} excitation energy density. (b) Results of the numeric image analysis where the coloured curves indicate the resulting traces of the numeric image analysis, along with the black dashed curves which represent the fit of their temporal profile based off the global fit done in (d). (c) Fit of the free spectral range in the energy region around the gain profile. (d) Numerically extracted lasing mode energies from all the lasing modes (color dots), with the time evolution of each mode shifted horizontally until all data sets fall on the same curve. Globally fit to all the data points is shown as the dashed curve.

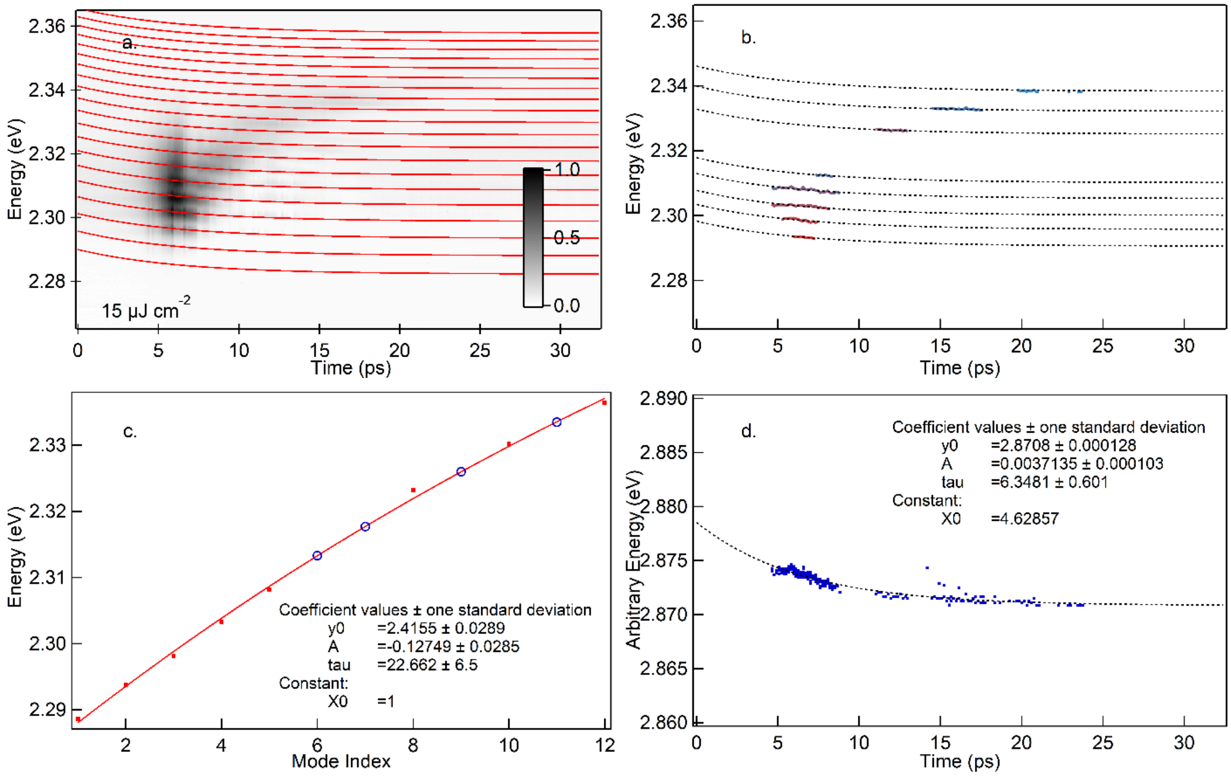

Supplementary Figure 6. Extracting laser gain profiles and mode energies. (a) Overlay of the numeric temporal photonic mode profiles with the lasing spectrum at 15 µJ cm^{-2} excitation energy density. (b) Results of the numeric image analysis where the coloured curves indicate the resulting traces of the numeric image analysis, along with the black dashed curves which represent the fit of their temporal profile based off the global fit done in (d). (c) Fit of the free spectral range in the energy region around the gain profile. (d) Numerically extracted lasing mode energies from all the lasing modes (color dots), with the time evolution of each mode shifted horizontally until all data sets fall on the same curve. Globally fit to all the data points is shown as the dashed curve.

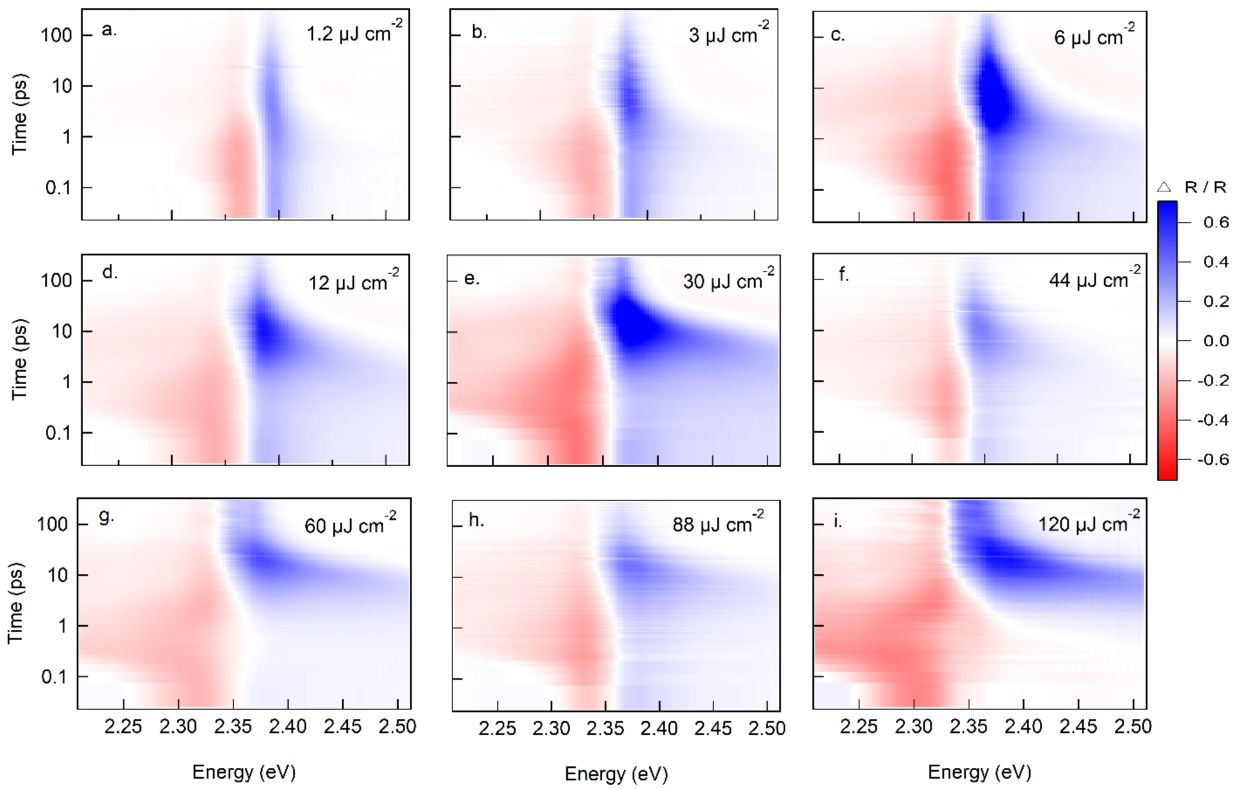

Supplementary Figure 7. Transient reflectance spectra. Complete set of ultrafast transient reflectance spectra at excitation photon energy of 3.1 eV and a sample temperature of 80 K. The range of excitation energy density spans from below lasing threshold (a), to the lasing threshold (b), lasing region (c) and (d), the saturation threshold (e), and the saturation region (f) - (i). Panels (a),(c),(e),and (i) are the same panels that appeared in Fig 4 in the main text.

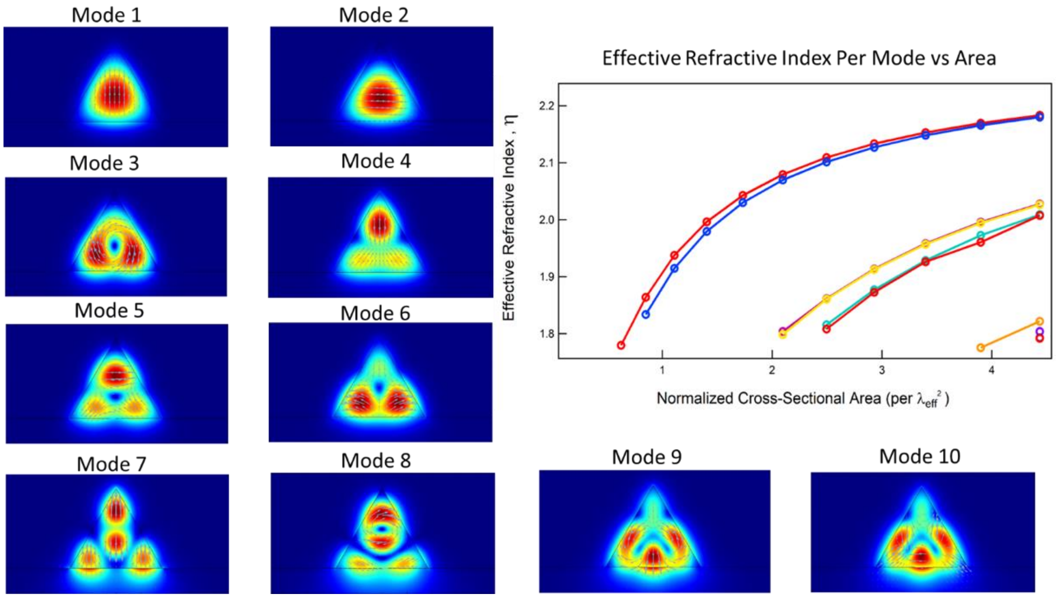

Supplementary Figure 8. Numerical simulation of wave-guiding modes in an idealized nanowire. We carried out the simulation using the finite difference method within the COMSOL package. The nanowire cross section was assumed to be an equilateral triangle, with a side-length of 400nm. An refractive index of n=2.3 was used for the $CsPbBr_3$ nanowire and n=1.765 for the sapphire substrate below the nanowire. At this size, there are ten optical modes supported, and their electric field profiles and polarizations (cyan vectors) are depicted in the images. Also shown are the effective refractive indexes as a function of nanowire cross sectional area, showing the increasing degree of confinement of the lowest-order modes as the nanowire size increases.

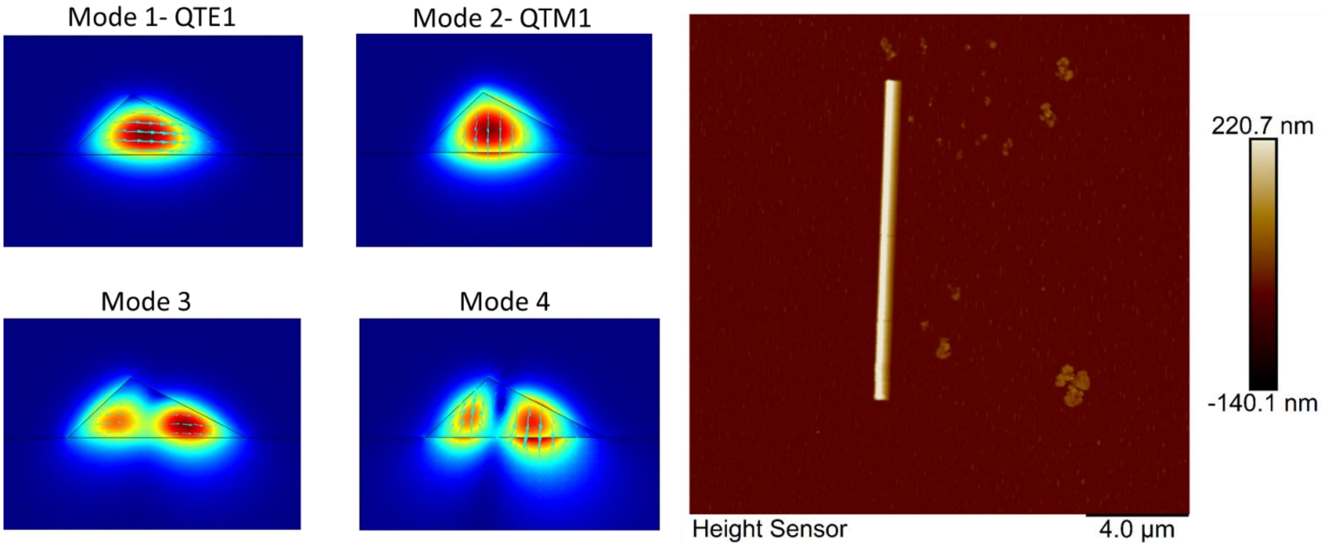

Supplementary Figure 9. Numerical simulation of wave-guiding modes in the actual nanowire. AFM image of the actual triangular nanowire is shown on the right. We carried out the finite difference simulation based on the experimental nanowire geometry. The first four solutions: quasi-transverse electric mode (Mode 1- QTE1), quasi-transverse magnetic mode (Mode 2-QTM1), higher order modes without trivial polarization (Mode 3 & Mode 4). Compared to the idealized equilateral triangular shape in Fig. S8, the lower symmetry in the modes results from the lower symmetry of the actual nanowire. The reduced spatial volume of the nanowire leads to more leaking out of the electric field. The fundamental optical mode (Mode 1) and is likely the mode responsible for lasing[1].

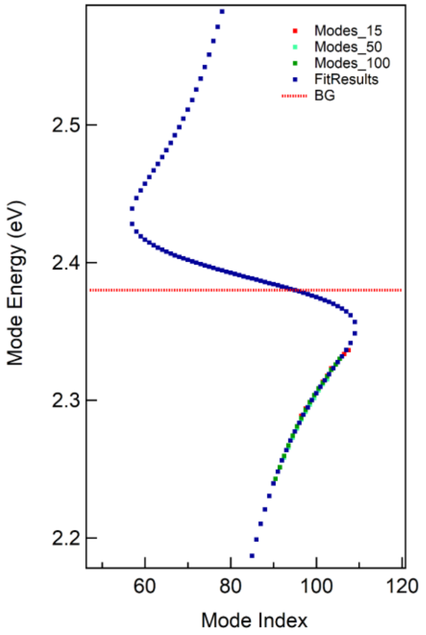

Supplementary Figure 10. Dielectric analysis of Fabry-Perot cavity modes. The dots are from the single Lorentzian oscillator model and other colored dots are experimental data points. The red-dotted line is resonant energy.

Supplementary Note 1. Data of lasing spectra from other representative NWs

For both nanowires presented in Supplementary Figure 1, we observe the same characteristics as those in Fig. 2 in the main text. These include the appearance of lasing threshold at excitation energy densities in μJ/cm^2 region, the saturation threshold at 10s μJ/cm^2, the narrowing followed

by blue-shift of gain profile with time, and the red-shift of each lasing mode with time. The change in spectral profile with nanowire length is determined by the Fabre-Perot cavity modes, equation (2) in main text. This leads to the inverse dependence of mode spacing on cavity length, $\Delta E = \frac{hC}{2n(\omega)} L^{-1}$, where h is Planck's constant, C is the speed of light, n is the refractive index (which depends on , and L is the length of the cavity (i.e., the nanowire). In agreement with this relationship, we find that mode spacing is smaller for longer L in Supplementary Figure 1.

The lasing spectral evolution from the microplate shows similar features as characteristic of that from the nanowires. These include the increasing spectral range and the red-shifting in the peak lasing position with excitation density in Supplementary Figure 2a, the initial narrowing followed by blue-shifting in laser gain profile with time in Supplementary Figure 2b&c, and the red-shifting of each mode with time or decreasing density in all three panels. For the microplate sample, the saturation threshold for lasing is not reached within the excitation density range investigated.

Supplementary Note 2. Sample lasing spectra from NWs at selected delay times

At excitation densities above the lasing thresholds, the narrowing of the lasing spectra takes longer time at higher initial excitation densities, Supplementary Figure 3a, b, c. A comparison of the initial broad lasing spectra at different excitation densities reveals the red-shift with carrier density, as determined by the density-dependent plasmon frequency, in agreement with excitation density dependent lasing spectra in Fig. 2 in the main text. The time integrated spectra in Supplementary Figure 3e illustrate the spectral congestion due to time-dependent red-shifts in each cavity mode attributed to changes in the refractive index with excitation density.

Supplementary Note 3. Quantitative determination of lasing mode energies

The dielectric function has real and imaginary components. Loss and gain are represented by positive and negative values respectively in the imaginary component. From our lasing profile we can extract the time dependent imaginary component of the dielectric function. To do this we use numeric image analysis, Supplementary Figure 4a, to track the spectral position of each laser mode over time coloured curves in Supplementary Figure 4b. The shifting of each mode is attributed to changes in the refractive index. Within experimental energy resolution, each lasing model can be

described by the same function of time. Using the overlap of different modes in the energy domain, we horizontally offset each so they all lay continuously head to tail, as shown as color dots in Supplementary Figure 4d. Then a single exponential fit is used to globally fit the time-dependent mode energies over the course of our experiment. Using the fit parameters from the global fit in Supplementary Figure 4d and the time offset (t_0) for each model, we fit each individual mode profile, dashed lines in Supplementary Figure 4b. From these individual fits, we obtain the intrinsic mode energies at the extrapolated low carrier density limit. These energies are depicted as red circles in Supplementary Figure 4c. The a few blue dots are those not well resolved in the experiment spectra and are interpolated from the fit function. Similar analysis is done for excitation energy densities at 50 μJ cm^{-2} (Supplementary Figure 5) and 15 μJ cm^{-2} (Supplementary Figure 6), respectively.

Supplementary Note 4. Numerical simulation of wave-guiding modes in nanowires

We carry out optical wave guide analysis in the nanowires using the finite-difference in the COMSOL software package. The analysis is done with the photon energy relevant to lasing, hν = 2.4 eV. We carry out simulation for an idealized nanowire of equilateral triangle cross section (Supplementary Figure 8) and the actual nanowire with the triangle cross section determined experimentally from atomic force microscopy (AFM) imaging. For the idealized nanowire, we used large cross sectional sizes of 5 μm to obtain ten optical modes, shown in Supplementary Figure 8. The number of supported modes decrease as the lateral sizes decrease, as shown in the plot of the dependence of effective refractive index on the cross sectional area. For the actual nanowire, we find four modes, shown in Supplementary Figure 9, along with the AFM image. Within the experimental energy window, we probe the two lowest energy modes (mode 1 and mode 2) that are nearly degenerate.

Supplementary Note 5. Chemical Potentials and Electronic Phase Transitions

We carry out numerical estimation of electronic phases (e.g. exciton/excitonic resonance of free carriers, non-degenerate electron-hole plasma, degenerate electron-hole plasma) using the methodology laid out in Versteegh et. al., PRB (2011)[2]. This method allows for a numerical

determination of the chemical potential, avoiding the erroneous high- and low-density limit expressions for the Mott density. This is achieved by using the definition of the carrier density, N, in terms of the chemical potential and temperature, for a bulk 3-D material:

$$N = \frac{1}{\pi^2}\left(\frac{2m_i}{\hbar^2}\right)\int_0^\infty \frac{\sqrt{E}}{e^{\frac{(E-\mu_i)}{k_b T}}+1} dE \quad \text{Supplementary Equation 1}$$

Here, i stands for index, which can be either the electron or the hole, m_i is the effective mass, taken from Becker et. al., Nature (2018)[3] (m_e=0.134 m_o, $m_h = 0.128 m_o$, $\epsilon_r = 4.8$), $\hbar$ is Planck's constant, k_b is Boltzmann's constant, T is temperature, and μ_i is the chemical potential. Supplementary Equation 1 is solved for both the electron and the hole effective masses, creating a mapping between temperature, electron (hole) chemical potential, and electron (hole) number densities. Next, these are used to calculate the plasma screening lengths, according to:

$$\lambda_{s,i} = \sqrt{\frac{\epsilon_o \epsilon_r}{e\hat{\ }2}}\sqrt{\frac{\partial \mu_i}{\partial n_i}} \quad \text{Supplementary Equation 2}$$

Here, $\lambda_{s,i}$ is the screening length of a single-component plasma, where once again the i stands for the index of either the electron or the hole, ϵ_o is the vacuum permittivity, ϵ_r is the static dielectric function[3], and e is the charge of a single electron. These are both solved, and then their results are combined so as to account for a two-component plasma;

$$\lambda_s^{-2} = \lambda_{s,e}^{-2} + \lambda_{s,h}^{-2} \quad \text{Supplementary Equation 3.}$$

Special care must be taken here so as to ensure that the number of electrons and holes are equal throughout, as they are in experiments. To this end, a map is created between the electron and hole densities, i.e. for a certain electron chemical potential, a corresponding hole chemical potential must be found such that there are equal electron and hole number densities. Then when taking the derivatives to generate the plasma screening length, this relationship must be obeyed to generate an accurate plasma screening length. The screening lengths are then compared against the unscreened exciton Bohr radius. The definition used here of the Mott density is the condition at which point the screening length of the plasma becomes shorter than the exciton Bohr radius; at this point, the coulomb potential between an unscreened electron-hole bound pair would be reduced to 1/e of its original value. It is at this density when the fraction of exciton or excitonic resonance rapidly approaches zero. Note that at temperatures above the thermal dissociation

energy (~ 340 K here), there are no excitons, even though the screening lengths are very long in the plasma; instead, free electrons in the conduction band is correlated with free holes in the valence band in the form of excitonic resonance in optical transition[4]. Above the Mott density, we refer to the system as either a non-degenerate or a degenerate electron-hole plasma (n-EHP, d-EHP). The distinction is formally where the de Broglie wavelengths of the electrons and holes are smaller than their inter-particle separations. This is approximately satisfied at the condition for population inversion. The final separation in the phase diagram is where the chemical potential becomes positive, i.e. population inversion is achieved, but we have designated the region by naming it the d-EHP region.

Note that the degenerate plasma is defined as where population inversion is achieved at the band edge, however through the introduction of mid-gap states, the requirements for population inversion may be fulfilled at lower carrier densities. The mechanism of the plasmon-assisted lasing is that a plasmon side-band appears in the electronic structure[5,6], easing the requirement to achieve population inversion.

Supplementary Note 6. Simulation of transient reflectance spectra

We simulate the reflectance spectrum using n, and k, as follows:

$$R(\omega) = \frac{((1-n(\omega))^2 + k(\omega)^2}{((1+n(\omega))^2 + k(\omega)^2} \text{ Supplementary Equation 4.}$$

Then a transient reflectance was generated using the following:

$$\Delta R(\omega) = \frac{R_2(\omega) - R_1(\omega)}{R_1(\omega)} \text{ Supplementary Equation 5.}$$

To simulate a representative shape for a plasmon reflectance, we chose $R_2(\omega)$ to have a plasmon frequency different from one, and for $R_1(\omega)$ to have no plasmon resonance ($\omega_p = 1$). To simulate a representative 'gain' feature, we changed $R_2(\omega)$ by replacing $k(\omega)^2$ throughout by - $k(\omega)^2$.

Supplementary Note 7. Nonlinear dispersion from the Lorentzian oscillator model

As we discuss in the main text, the nonlinear dispersion with negative curvature can be attributed to the energy-dependent refractive index, equations 2-4. In our analysis, we found the experimental data to lie on the computed mode profile with $\hbar\omega_o = 2.38$ eV, $\Gamma = 72$ meV, and $\hbar\omega_p = 700$ meV. Shown below is the mode profile for these conditions, and also the experimental data points whose mode indexes have been shifted to lie on the curve. The figure in the main paper is the same, except centered closer around the experimental points. It is important to point out that the single Lorentzian oscillator model is meant as a qualitative illustration of how the experimental mode energy dispersion can be described. We do not attempt any quantitative interpretation of the parameters. In reality, the energy-dependent dielectric function of single crystal CsPbBr3 nanowires at excitation densities above the Mott density is more complex.

Note that when the dephasing of the oscillator tends toward zero, a mode profile reminiscent of upper and lower polariton branches is retrieved. This suggests that lasing in a material with a resonance should be expected to have a negative dispersion for the lasing peaks, but that this does not necessarily correspond to polaritons.

Supplementary References

1. Maslov, A. V & Ning, C. Z. Reflection of guided modes in a semiconductor nanowire laser. *Cit. Appl. Phys. Lett* **83,** 1237 (2003).

2. Versteegh, M. A. M., Kuis, T., Stoof, H. T. C. & Dijkhuis, J. I. Ultrafast screening and carrier dynamics in ZnO: Theory and experiment. *Phys. Rev. B - Condens. Matter Mater. Phys.* **84,** (2011).

3. Becker, M. A. *et al.* Bright triplet excitons in caesium lead halide perovskites. *Nature* **553,** 189–193 (2018).

4. Koch, S. W., Kira, M., Khitrova, G. & Gibbs, H. M. Semiconductor excitons in new light. *Nat. Mater.* **5,** 523–531 (2006).

5. Vigil-Fowler, D., Louie, S. G. & Lischner, J. Dispersion and line shape of plasmon satellites in one, two, and three dimensions. *Phys. Rev. B* **93,** 235446 (2016).

6. Caruso, F. & Giustino, F. Theory of electron-plasmon coupling in semiconductors. *Phys. Rev. B* **94,** 115208 (2016).

7. Haug, H. & Koch, S. W. *Quantum Theory of the Optical and Electronic Properties of Semiconductors*. (WORLD SCIENTIFIC, 2009). doi:10.1142/7184

8. Miyata, K. *et al.* Large Polarons in Lead Halide Perovskites. *Sci. Adv.* **3,** e1701217 (2017).

9. Boyd, R. W. *Nonlinear Optics*. (Academic Press, 2008).
